# Alpine meadow degradation reshapes diazotroph community assembly and enhances putative recruitment of diazotrophs from legume soils to sedge soils

**DOI:** 10.3389/fmicb.2026.1883674

**Published:** 2026-08-19

**Authors:** Qinen He, Yingcheng Wang, Zhen Wang, Huifang Shi, Guangxin Lu, Huakun Zhou

**Affiliations:** 1 College of Animal of Science and Veterinary, Qinghai University, Xining, China; 2 Qinghai Provincial Key Laboratory of Restoration Ecology in Cold Regions, Northwest Institute of Plateau Biology, Chinese Academy of Sciences, Xining, China; 3 College of Ecological and Environmental Engineering, Qinghai University, Xining, China; 4 College of Forestry and Grassland, Qinghai University, Xining, China

**Keywords:** aboveground biomass, alpine meadow degradation, community assembly, diazotrophs, nifH gene, soil microorganisms

## Abstract

**Introduction:**

Biological nitrogen fixation is a key microbial process sustaining nutrient cycling and plant productivity in nitrogen-limited alpine meadows. However, how alpine meadow degradation reshapes diazotrophic community composition and assembly across different plant species, and how these changes relate to aboveground biomass, remain poorly understood.

**Methods:**

We used the nifH gene as a molecular marker to investigate diazotrophic communities in rhizosphere and bulk soils associated with the legume *Oxytropis ochrocephala*, and the sedge *Carex alatauensis* in undegraded and degraded alpine meadows on the Qinghai-Tibet Plateau.

**Results:**

Meadow degradation markedly reduced diazotroph *α*-diversity and shifted community composition, with *Proteobacteria*, *Cyanobacteria*, *Firmicutes*, and *Actinobacteriota* dominating the assemblages. Diazotrophic communities in legume soils were primarily structured by deterministic processes, whereas those in sedge soils were more strongly influenced by stochastic processes. Source-tracking analysis suggested that legume soils may serve as an important reservoir of diazotrophs for sedge soils, with the potential contribution increasing under degradation. Exploratory PCA-based piecewise SEM indicated that degradation mainly affected soil nutrient status and diazotroph *β*-diversity, while aboveground biomass was only weakly mediated by the soil nutrient–diazotroph–vegetation diversity pathway.

**Discussion:**

These findings indicate that alpine meadow degradation reorganizes diazotrophic diversity, community assembly, and potential source contributions between legume and sedge soils. Legume soils may therefore represent an important diazotroph reservoir for sedge-associated soils during alpine meadow degradation.

## 1 Introduction

The Qinghai-Tibet Plateau (QTP), the highest plateau in the world, is dominated by grassland ecosystems that not only support regional livestock husbandry but also provide crucial ecological services (Cheng and Wu, 2007; Borer et al., 2014; Raiesi and Riahi, 2014). However, grassland degradation caused by factors such as climate change and human activities now poses a serious threat to the region’s biodiversity and ecological security (Su et al., 2006; Li et al., 2016; Wang et al., 2018; Xu et al., 2020). Alpine meadows are a typical grassland ecosystem on the QTP (Gao et al., 2014). In recent years, alpine meadows on the QTP have experienced severe degradation, particularly common in the Sanjiangyuan area of the central QTP (Sun et al., 2013; Shao et al., 2017). Changes in soil microbial communities can serve as important biological indicators for assessing the process and status of grassland degradation (Kardol and Wardle, 2010; Singh, 2015). Microorganisms capable of directly utilizing atmospheric N are known as diazotrophs. They primarily consist of prokaryotes, including bacteria and archaea (Dos Santos et al., 2012). Currently, there is limited research on diazotrophs in the context of alpine meadow degradation. Therefore, studying the structure and diversity of soil diazotrophic communities in alpine meadows and their response to degradation holds significant scientific importance.

Soil nitrogen (N) is a fundamental nutrient regulating plant growth and ecosystem functioning in terrestrial ecosystems (Xu et al., 2015). In grassland ecosystems, N availability often acts as a key limiting factor for vegetation restoration and the maintenance of primary productivity (Bai et al., 2010). Nitrogen cycling is a globally significant biogeochemical process, almost entirely driven by environmental microorganisms (Kuypers et al., 2018), with nitrogen fixation serving as a pivotal process within the microbial nitrogen cycle (Levy-Booth et al., 2014). Diazotrophic microbes are key contributors to the nitrogen cycle in grassland ecosystems and are essential for their long-term stability and sustainable development (Nshimiyimana et al., 2024). During grassland degradation, legume may act as keystone plant species by alleviating soil nitrogen limitation through their associations with diazotrophic microorganisms. This process can enhance nitrogen inputs, help maintain grassland productivity, and contribute to nitrogen cycling in grassland ecosystems (van der Heijden et al., 2008; Vitousek et al., 2013). In alpine meadows with high soil moisture, sedge are the absolute dominant and constructive species, playing a crucial role in maintaining the structural and functional stability of fragile ecosystems (Yang et al., 2013). Currently, the nitrogen-fixing capacity of legume has been extensively studied. However, it remains unclear whether and how sedge participate in or influence nitrogen fixation processes during the degradation of alpine meadows, as systematic studies on this topic are currently lacking. Therefore, conducting an in-depth investigation into the diazotrophic communities associated with legume and sedge during the degradation of alpine meadows is of significant importance for gaining a deeper understanding of the nitrogen cycle in grassland ecosystems.

Microbial community assembly refers to the process by which species from a regional species pool immigrate, establish, and persist, thereby colonizing and shaping the local microbial community through interactive processes (Zhou and Ning, 2017). Two mainstream theoretical frameworks, niche-based theory and neutral process theory, have been widely used to explain the assembly of soil microbial communities (Chave, 2004; Dumbrell et al., 2010; Wang et al., 2013). Niche-based theory emphasizes deterministic processes, in which community composition is shaped by environmental filtering and biotic interactions. Environmental filtering is often associated with abiotic factors such as soil pH, nutrient availability, and moisture, whereas biotic interactions include positive and negative interactions among taxa (Fierer and Jackson, 2006; Chase and Myers, 2011). In contrast, neutral process theory highlights stochastic processes, such as ecological drift, dispersal limitation, and random birth-death events, under the assumption of ecological equivalence among taxa (Chave, 2004). Increasing evidence suggests that microbial community assembly is not governed by a single mechanism, but is jointly shaped by deterministic and stochastic processes (Dumbrell et al., 2010; Wang et al., 2013). In contrast, stochastic processes encompass dispersal limitation, ecological drift, and stochastic birth-death events (Chase and Myers, 2011). Community assembly processes are strongly influenced by abiotic factors such as soil carbon and nitrogen (Zhou et al., 2022; Kong et al., 2022), leading to significant differences in microbial assembly processes across habitats. For instance, stochastic processes are less critical in forest soils, while dispersal limitation dominates bacterial assembly in temperate grasslands (Chen et al., 2023). Previous studies investigated the assembly processes of bacterial communities near the roots of different shrubs on the Tibetan Plateau and found that both habitat changes and shrub species identity increased bacterial community heterogeneity, resulting in community differentiation (Qu et al., 2023). Shrub species strongly induced bacterial community and strongly affected its selection, while habitat heterogeneity decreased its dispersal ability. However, the role of deterministic and stochastic processes in assembling diazotrophic community in both rhizosphere and bulk soils across different plant species during alpine meadow degradation has not been fully elucidated. Therefore, in this study we hypothesize that: (i) alpine meadow degradation alters the diversity and assembly of diazotrophic community in legume and sedge; (ii) meadow degradation increases the potential contribution of legume-soil diazotrophs to sedge soils; (iii) degradation-induced changes in diazotrophic communities are linked to vegetation attributes and may have indirect implications for aboveground biomass.

## 2 Materials and methods

### 2.1 Study area

**Figure 1:**
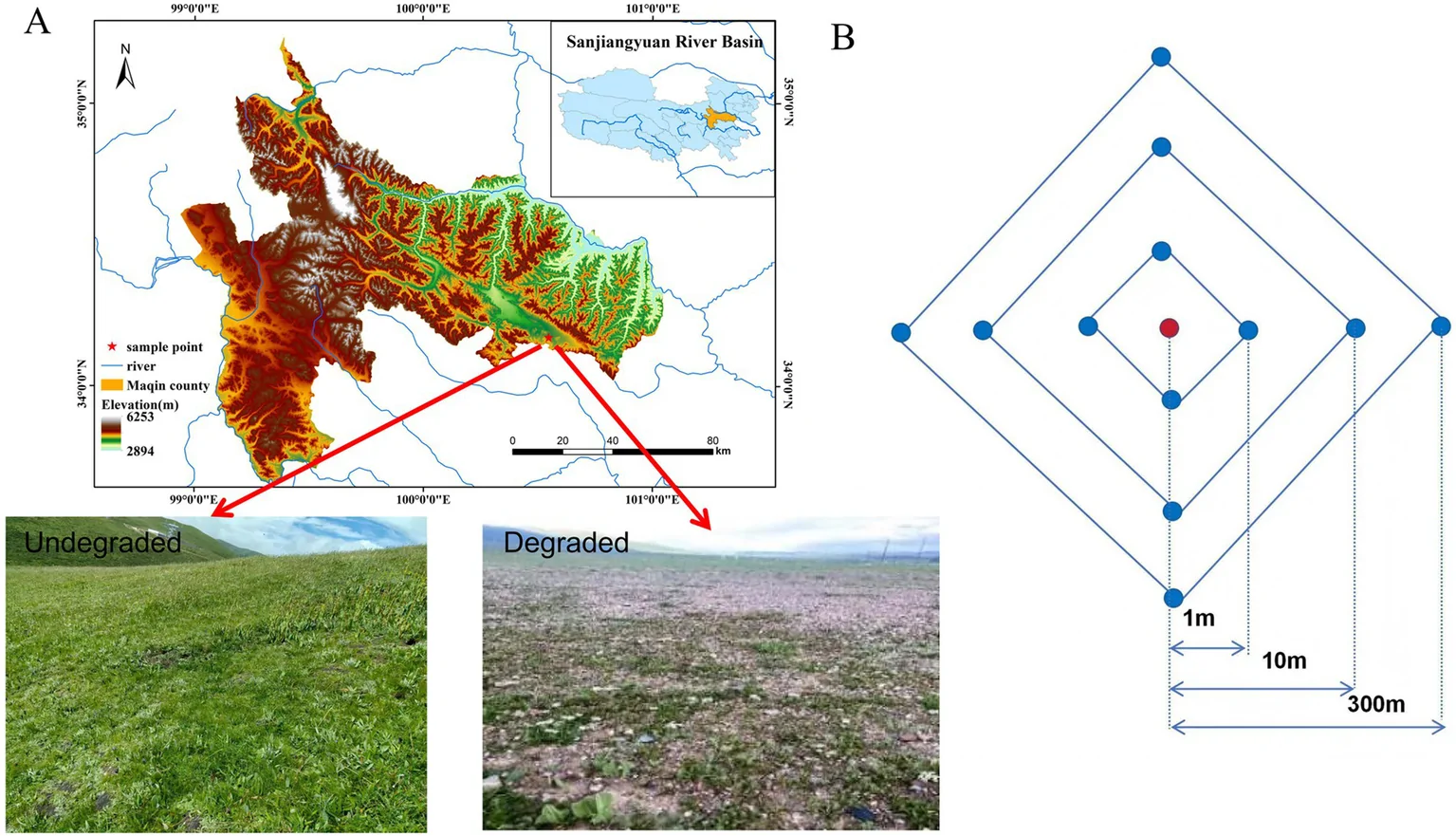
Location of the study and the sampling point **(A)** and sampling plan **(B)**.

Samples for this study were collected during the summer of 2024 at the “Sanjiangyuan Grassland Ecosystem National Field Science Observation and Research Station” located in Dawu Town, Maqin County, Golog Tibetan Autonomous Prefecture, Qinghai Province. The region features a plateau continental climate, with the soil types predominantly consisting of alpine meadow soil and alpine shrub meadow soil. The grasslands are primarily characterized by alpine meadows and alpine wetlands, with a small distribution of alpine steppe. According to the grassland degradation classification criteria proposed by Ma Yushou, we selected undegraded and degraded alpine meadows (Ma et al., 2002) (Figure 1A). The undegraded site has an average elevation of 4,093 m, located at 34°20′19″N, 100°28′03″E, with well-developed vegetation and coverage exceeding 80%; the degraded site has an average elevation of 3,990 m, located at 34°19′59″N, 100°29′53″E, it exhibits low vegetation coverage of around 50%, with prominent bare soil patches and rodent mounds, and is under considerable grazing pressure. The dominant vegetation are *Carex alatauensis*, *Carex capillifolia*, *Kobresia pygmaea*, *Oxytropis ochrocephala*, *Saussurea superba*, *Leontopodium nanum.*

### 2.2 Soil and plant sampling

Samples were collected using a nested sampling design, where smaller sampling plots are nested within larger ones. In this study, using the central point as a reference, thirteen 50 × 50 cm sampling plots were systematically arranged in a perpendicular transect pattern at distances of 1 m, 10 m, and 300 m from the sampling center (Figure 1B). In this study, legume (*Oxytropis ochrocephala*) and sedge (*Carex alatauensis*) were selected through field investigations. Within each quadrat, three individuals of each selected plant species were carefully excavated with intact root systems using a shovel, and the soil adhering to their roots was retained (Cao et al., 2019), the three individuals were then pooled as one biological replicate. In this study, bulk soil was defined as the soil shaken off from the roots, whereas rhizosphere soil was defined as the soil tightly adhering to the roots that could not be removed by shaking (Chaudhary et al., 2015). Rhizosphere and bulk soil collected from the root systems were used for DNA extraction. In parallel, two random soil cores from the surface layer (0–15 cm) were collected within each quadrat using a soil auger. These cores were homogenized into one composite sample, placed into pre-numbered sample bags, and transported to the laboratory for subsequent soil physicochemical analyses. The measured soil physicochemical parameters were used as environmental variables corresponding to the microbial samples collected from the same quadrat. A total of 104 samples were collected in this study (2 degradation gradients × 2 species × 2 niches × 13 replicates). Aboveground biomass (AGB) was used as a proxy for grass productivity. In each 50 × 50 cm quadrat, all aboveground plant material was clipped at ground level, placed in paper bags, oven-dried at 65 °C to constant weight, weighed, and converted to area-based dry biomass. Detailed measurements of the physical and chemical properties of the soils are provided in the Supplementary materials.

### 2.3 DNA extractions and PCR

Total genomic DNA was extracted from 0.5 g of each soil sample using the DNeasy PowerSoil Pro Kit (250). The *nifH* gene was amplified using the primers nifHF (5′-AAAGGYGGWATCGGYAARTCCACCAC-3′) and nifHR (5′-TTGTTSGCSGCRTACATSGCCATCAT-3′). The resulting DNA amplicons were subsequently preserved by freezing, transported under cold chain conditions, and sent to Majorbio Gene Technology Co., Ltd. (Shanghai, China) for amplicon sequencing. The constructed amplicon libraries are subjected to Nextseq 2000 sequencing on either the Illumina platform.

### 2.4 Processing of sequence data

Raw FASTQ files were demultiplexed according to sample-specific barcodes using an in-house Perl script. Barcodes and primer sequences were removed, and low-quality reads were filtered using fastp v0.19.6. Paired-end reads were merged using FLASH v1.2.7 based on their overlapping regions. The quality-filtered merged sequences were dereplicated, sorted by abundance, and denoised using the UNOISE algorithm to generate zero-radius operational taxonomic units (ZOTUs). Chimeric sequences were identified and removed during the denoising procedure. Taxonomic identity was determined using the RDP classifier (Version 2.2, http://rdp.Cme.msu.Edu/) in the FunGene (Version 9.6, http://fungene.cme.Msu.edu/) database. To minimize the effect of uneven sequencing depth among samples, the ZOTU table was rarefied to 16,675 sequences per sample for the nifH gene. The rarefied ZOTU table was used for subsequent analyses of diazotrophic community diversity, composition, and assembly processes.

### 2.5 Statistical analysis

Margalef index, Shannon index (*H*), Simpson index (*H*′), and Pielou index (*E*) were selected to estimate the community species diversity (Wen et al., 2020).

$$\begin{array}{l} H^{\prime }=1-\sum_{i=1}^{S}p_{i}^{2}\;H=-\sum_{i=1}^{S}p_{i}\ln(p_{i})\;E=\frac{H}{\ln(S)}\;\\ p_{i}=\frac{IV_{i}}{IV_{\text{total}}}\;IV_{i}=\frac{\left(rh+\mathrm{ra}+\mathrm{rc}\right)}{3}\times 100\% \end{array}$$

Where *IV_i_* is the importance value of the plant species *i*, *rh*, *ra*, and *rc* are the proportion of the coverage, abundance, and height of species *i* to the total of all species in plot, respectively. *Pi* is the relative importance value of species *i* in each plot, and *S* is the number of plant species in each plot.

Analysis of variance (ANOVA) and independent samples t-tests were used to assess statistical differences in vegetation and soil property indices. A significance threshold of *p* < 0.05 was applied to determine significant differences among groups. Results are expressed as mean ± standard error (SE). Beta diversity was assessed using principal coordinate analysis (PCoA) based on Bray–Curtis dissimilarity matrices. The statistical significance of differences between sample groups was further tested using permutational multivariate analysis of variance (PERMANOVA) on Bray–Curtis dissimilarity matrices. The normalized stochasticity ratio (NST) was used to quantify the relative importance of deterministic and stochastic processes in diazotrophic community assembly (Ning et al., 2019). NST values range from 0 to 1, with values < 0.5 indicating a greater contribution of deterministic processes and values > 0.5 indicating a greater contribution of stochastic processes. To further identify the specific ecological processes underlying community assembly, the iCAMP package was used to partition assembly processes into homogeneous selection, heterogeneous selection, dispersal limitation, homogenizing dispersal, and drift processes. In iCAMP analysis, beta net relatedness index (*β*NRI) was first used to evaluate selection processes. Pairwise comparisons with βNRI < −1.96 were classified as homogeneous selection, whereas those with βNRI > + 1.96 were classified as heterogeneous selection. For comparisons with |βNRI| ≤ 1.96, the modified Raup–Crick metric (RC) was used to distinguish stochastic processes: RC > +0.95 was classified as dispersal limitation, RC < −0.95 as homogenizing dispersal, and |RC| ≤ 0.95 as drift processes (Ning et al., 2020). Homogeneous selection and heterogeneous selection were grouped as deterministic processes, whereas dispersal limitation, homogenizing dispersal, and drift processes were grouped as stochastic processes.

Exploratory piecewise structural equation modeling (piecewiseSEM) was performed using the “piecewiseSEM” R package to examine potential direct and indirect pathways linking alpine meadow degradation, soil nutrient status, diazotroph β-diversity, vegetation diversity, and aboveground biomass (AGB). To avoid circularity in composite-variable construction, soil nutrient status and vegetation diversity were represented by PCA-derived PC1 scores based on soil nutrient variables and vegetation diversity indices, respectively. This analysis was used to evaluate whether degradation-induced changes in soil nutrients and diazotrophic communities were associated with variation in AGB. Model fit was evaluated using Fisher’s C statistic, with a non-significant *p* value (*p* > 0.05) indicating an adequate overall model fit. The Akaike information criterion (AIC) was also used to compare alternative models, with lower AIC values indicating better model support. To construct a model tracing the sources of soil diazotrophs across different plant species, we utilized the SourceTracker tool on the Galaxy platform.^1^ Within this framework, the target communities to be evaluated were defined as the “sink” (sedge), and the potential contributing diazotroph communities were defined as the “source” (legume). Based on the community structures of the source and sink samples, the proportions of the sink communities derived from each source were predicted. Subsequently, the mean potential contribution ratios of diazotrophs from different sources to rhizosphere and bulk soil compartments were calculated according to the SourceTracker output. Finally, a schematic diagram illustrating the model of soil diazotroph sources was generated. All statistical analyses and data visualizations were performed using R software (version 4.5.0).

## 3 Results

### 3.1 Meadow degradation significantly alters both the vegetation community and soil properties

Meadow degradation significantly alters the vegetation community structure and soil properties. Compared to undegraded meadows, degradation significantly reduced Margalef index and vegetation Cover. However, no significant differences were observed in the Simpson index, Shannon index, and Pielou index (Table 1). Degradation significantly increased soil pH and Available potassium (AK), but significantly decreased Soil organic carbon (SOC), Total nitrogen (TN), Total phosphorus (TP), Soil organic matter (SOM), and soil microbial biomass indices. NO_3_^−^-N, NH_4_^+^-N, and Available phosphorus (AP) showed decreasing trends but did not differ significantly between undegraded and degraded meadows (Table 1). These results indicate that meadow degradation leads to simplified plant communities and a pronounced decline in soil nutrient availability.

**Table 1:** Comparison of vegetation and soil characteristics between undegraded and degraded alpine meadows.

Indicator	Undegraded	Degraded	*p*
Margalef index	1.70 ± 0.15	1.34 ± 0.07	0.036*
Simpson index	0.82 ± 0.01	0.79 ± 0.01	0.321
Shannon index	0.56 ± 0.01	0.59 ± 0.01	0.083
Pielou index	0.29 ± 0.02	0.32 ± 0.01	0.215
Vegetation cover	92.15 ± 2.57	50.00 ± 0.00	<0.001***
Vegetation height	4.44 ± 0.34	5.63 ± 0.76	0.169
Species	8.08 ± 0.65	6.85 ± 0.27	0.092
Fresh biomass	92.02 ± 10.54	100.66 ± 12.17	0.597
Aboveground dry biomass	21.03 ± 1.96	29.83 ± 2.83	0.017*
Soil pH	6.67 ± 0.04	7.83 ± 0.04	<0.001***
Soil temperature	18.08 ± 0.66	16.47 ± 0.59	0.082
Soil moisture	90.90 ± 0.63	74.71 ± 3.78	<0.001***
Soil organic carbon	56.9 ± 7.4	34.6 ± 2.1	0.008**
Total nitrogen	9.0 ± 0.3	3.9 ± 0.3	<0.001***
Total phosphorus	0.9 ± 0.0	0.8 ± 0.0	0.04*
NO_3_^−^-N	86.98 ± 6.14	71.5 ± 4.60	0.055
NH_4_^+^-N	22.59 ± 3.37	15.48 ± 2.54	0.104
Available phosphorus	4.28 ± 0.39	4.21 ± 0.19	0.855
Available potassium	77.75 ± 3.15	138.36 ± 12.64	<0.001***
Soil organic matter	98.1 ± 12.73	59.6 ± 3.68	0.007**
Microbial biomass nitrogen	160.85 ± 8.96	87.95 ± 5.85	<0.001***
Microbial biomass phosphorus	183.06 ± 46.66	46.97 ± 4.42	0.01*
Microbial biomass carbon	1042.86 ± 52.68	566.41 ± 30.27	<0.001***

*, **, and *** indicate *p* < 0.05, *p* < 0.01, and *p* < 0.001, respectively.

### 3.2 Diversity and composition of diazotrophic communities in soils of degraded alpine meadows

**Figure 2:**
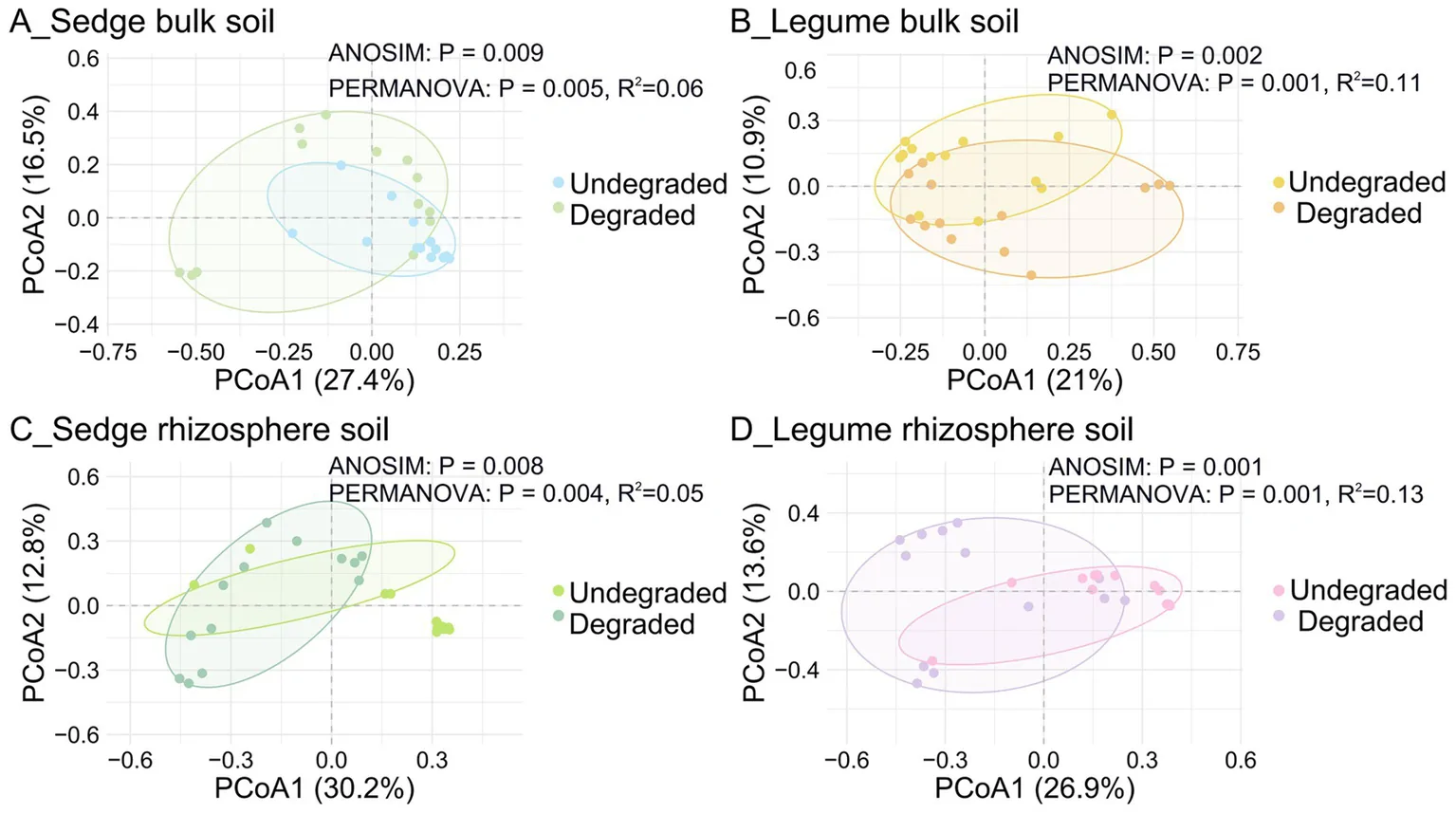
Effects of alpine meadow degradation on the structure (based on Bray-Curtis distance) of diazotrophic communities. **(A)** The PCoA of diazotrophic communities associated with sedge in bulk soil; **(B)** The PCoA of diazotrophic communities associated with legume in bulk soil; **(C)** The PCoA of diazotrophic communities associated with sedge in rhizosphere soil; **(D)** The PCoA of diazotrophic communities associated with legume in rhizosphere soil.

The *α*-diversity, as measured by ACE, Chao1, Richness, Pielou, and Shannon indices, was generally affected by meadow degradation. We found that undegraded meadows exhibited significantly higher α-diversity than degraded meadows, while no significant differences were observed between plant species (legume *vs.* sedge) or between rhizosphere and bulk soils under the same degradation status (Table 2). Notably, in undegraded meadows, Pielou and Shannon indices differed significantly between legume and sedge, whereas in degraded meadows, Pielou differed significantly between rhizosphere and bulk soil for legume (Table 2). These results indicate that meadow degradation is the main driver of diazotrophic communities α-diversity, while different plant species and soil compartment generally have minor effects. PCoA analysis indicated that degraded and undegraded samples showed distinct spatial separation (Figures 2A–D). PERMANOVA further showed that diazotrophic community composition was significantly affected by degradation status (*R*^2^ = 0.090, *F* = 10.44, *p* = 0.0001), plant type (*R*^2^ = 0.027, *F* = 3.165, *p* = 0.002), and soil compartment (rhizosphere vs. bulk soil; *R*^2^ = 0.016, *F* = 1.832, *p* = 0.0391). Among these factors, degradation status explained the largest proportion of community variation (Supplementary Table S2).

**Table 2:** Alpha diversity of the diazotrophic community.

Degradation	Group	ACE	Chao1	Richness	Pielou	Shannon
Undegraded	Legume bulk soil	361.77 ± 51.85a	358.54 ± 48.90a	276.38 ± 37.68a	0.64 ± 0.02a	3.58 ± 0.17a
Undegraded	Sedge bulk soil	396.70 ± 55.30a	372.39 ± 48.88a	299.23 ± 43.84a	0.56 ± 0.03a	3.16 ± 0.21a
Undegraded	Legume rhizosphere soil	351.03 ± 43.25a	344.68 ± 45.09a	282.46 ± 35.85a	0.63 ± 0.02a^A^	3.51 ± 0.18a^A^
Undegraded	Sedge rhizosphere soil	304.83 ± 50.88a	301.92 ± 49.49a	243.38 ± 41.63a	0.49 ± 0.03a^B^	2.70 ± 0.26a^B^
Degraded	Legume bulk soil	237.94 ± 49.95b	222.03 ± 50.07b	182.08 ± 39.90b	0.55 ± 0.03a^1^	2.77 ± 0.24b
Degraded	Sedge bulk soil	214.50 ± 21.72b	204.52 ± 18.46b	158.08 ± 14.11b	0.56 ± 0.03a	2.78 ± 0.14a
Degraded	Legume rhizosphere soil	234.33 ± 33.57b	229.30 ± 33.42b	186.62 ± 29.25b	0.44 ± 0.02b^2^	2.27 ± 0.15b
Degraded	Sedge rhizosphere soil	275.52 ± 35.83a	266.02 ± 35.61a	213.38 ± 28.99a	0.47 ± 0.03a	2.49 ± 0.21a

Different lowercase letters indicate significant differences between the degraded and undegraded sites for the same species; Different uppercase letters indicate significant differences between different species under the same degradation gradient; different numbers indicate significant differences between rhizosphere and bulk soil for the same species under the same degradation gradient (*n* = 13).

**Figure 3:**
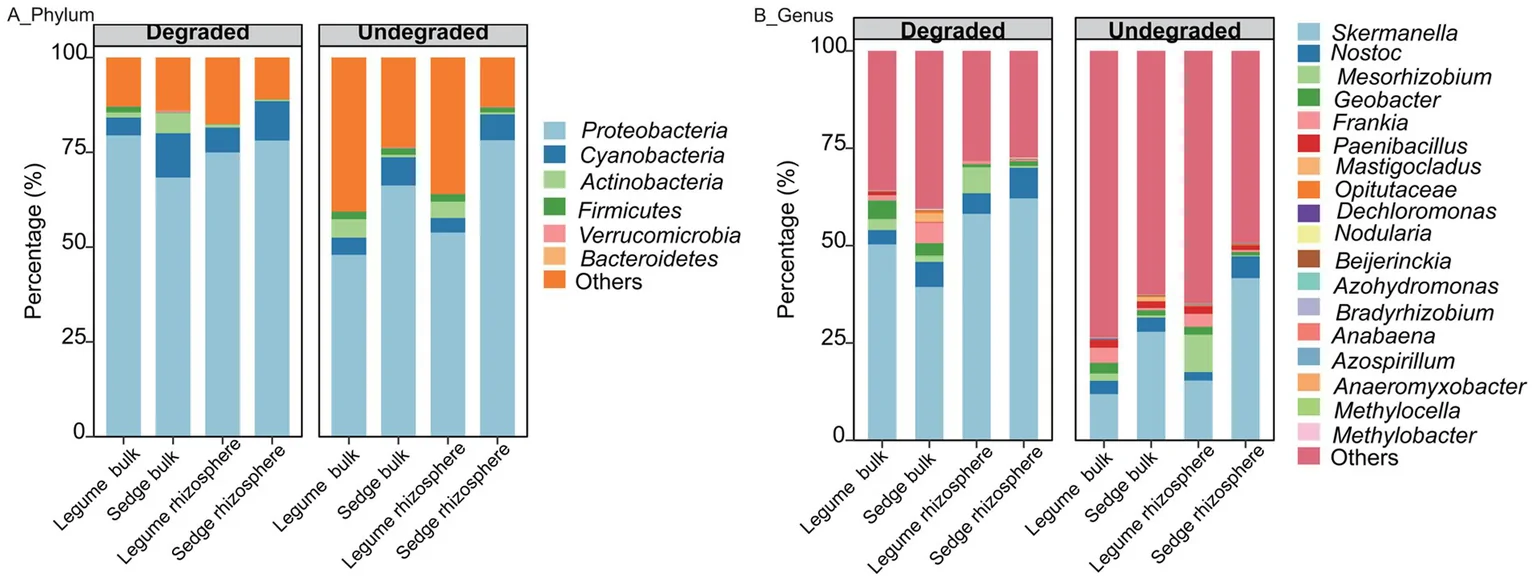
Composition of diazotrophs in undegraded and degraded alpine grassland soils. **(A)** Soil diazotrophic communities composition at the phylum level; **(B)** Soil diazotrophic communities composition at the genus level.

Taxonomic annotation of the diazotrophic communities in both rhizosphere and bulk soils for the two plant species was conducted at the phylum and genus levels. The results at the phylum level revealed that *Proteobacteria*, *Cyanobacteria*, *Firmicutes*, and *Actinobacteriota* were the dominant phyla (Figure 3A). The relative abundance of *Proteobacteria* was significantly higher in both the rhizosphere and bulk soils of legume in degraded meadows compared to undegraded meadows (rhizosphere: 74.96% *vs.* 53.84%; bulk: 79.45% *vs.* 47.98%, respectively). In contrast, no significant difference was observed for sedge. At the genus level, *Skermanella*, *Nostoc*, *Mesorhizobium*, *Geobacter*, *Frankia*, and *Paenibacillus* were predominant (Figure 3B). Among these, *Skermanella* and *Nostoc* showed higher relative abundance in the rhizosphere soils of degraded meadows, with significantly higher abundance in sedge compared to legume (Supplementary Figure S1). *Mesorhizobium* was more abundant in the rhizosphere of legume, but its abundance decreased under degraded conditions. In contrast, *Frankia* and *Paenibacillus* were more abundant in legume from undegraded meadows (Supplementary Table S1). These findings indicate that meadow degradation is the primary driver shaping diazotrophic community composition, while different plant species (sedge *vs.* legume) and soil compartment (rhizosphere soil *vs*. bulk soil) also influence the distribution of specific nitrogen-fixing genera.

### 3.3 Assembly processes of diazotrophic communities across different plant species and under meadow degradation

**Figure 4:**
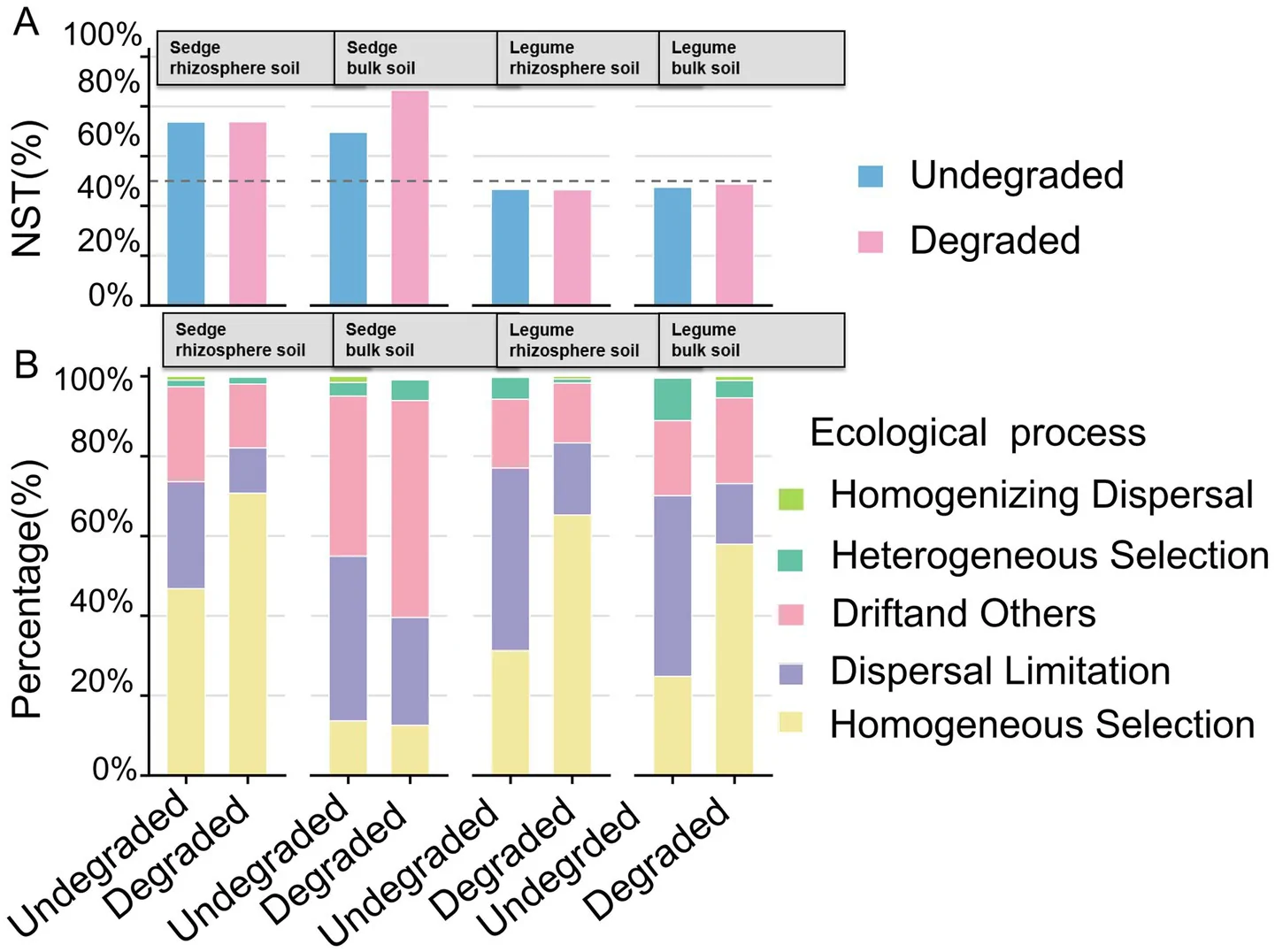
Assembly process of diazotrophs community. **(A)** NST values of diazotrophic community in degraded and undegraded alpine meadows; **(B)** The assembly process of diazotrophic community in degraded and undegraded alpine meadows based on iCAMP measurement.

We used iCAMP and NST to disentangle the differences in the assembly mechanisms governing the soil diazotrophic communities (Figure 4). Based on the NST analysis, diazotrophic communities associated with legume in both undegraded and degraded meadows were predominantly governed by deterministic processes, indicating that legume consistently exert strong host selection on diazotrophic community assembly regardless of meadow degradation. In contrast, diazotrophic communities associated with sedge in both undegraded and degraded meadows were mainly driven by stochastic processes (Figure 4A), reflecting differences in diazotroph community assembly patterns among different plant species along the grassland degradation gradient. The iCAMP analysis further quantified the assembly processes of diazotrophic communities in undegraded and degraded meadows. Legume-associated diazotrophic communities showed a relatively stronger contribution of deterministic processes, as reflected by the combined contribution of homogeneous and heterogeneous selection. However, the dominant assembly component shifted with meadow degradation, from a greater contribution of dispersal limitation in undegraded legume soils to a stronger contribution of homogeneous selection in degraded legume soils (Figure 4B). This result indicates that meadow degradation does not weaken the deterministic regulation of diazotrophic communities by legume, but instead alters their assembly mechanisms by strengthening environmental filtering. Interestingly, diazotrophic communities in the rhizosphere of sedge were dominated by homogeneous selection in both undegraded and degraded meadows, whereas diazotrophic communities in bulk soil were mainly governed by dispersal limitation and ecological drift (Figure 4B). These findings suggest that although diazotrophic community assembly associated with sedge is overall dominated by stochastic processes, consistent environmental filtering operates within the rhizosphere, while stochastic processes such as dispersal limitation and drift primarily shape diazotrophic communities in bulk soil.

### 3.4 Meadow degradation drives the putative recruitment of diazotrophs by sedge

**Figure 5:**
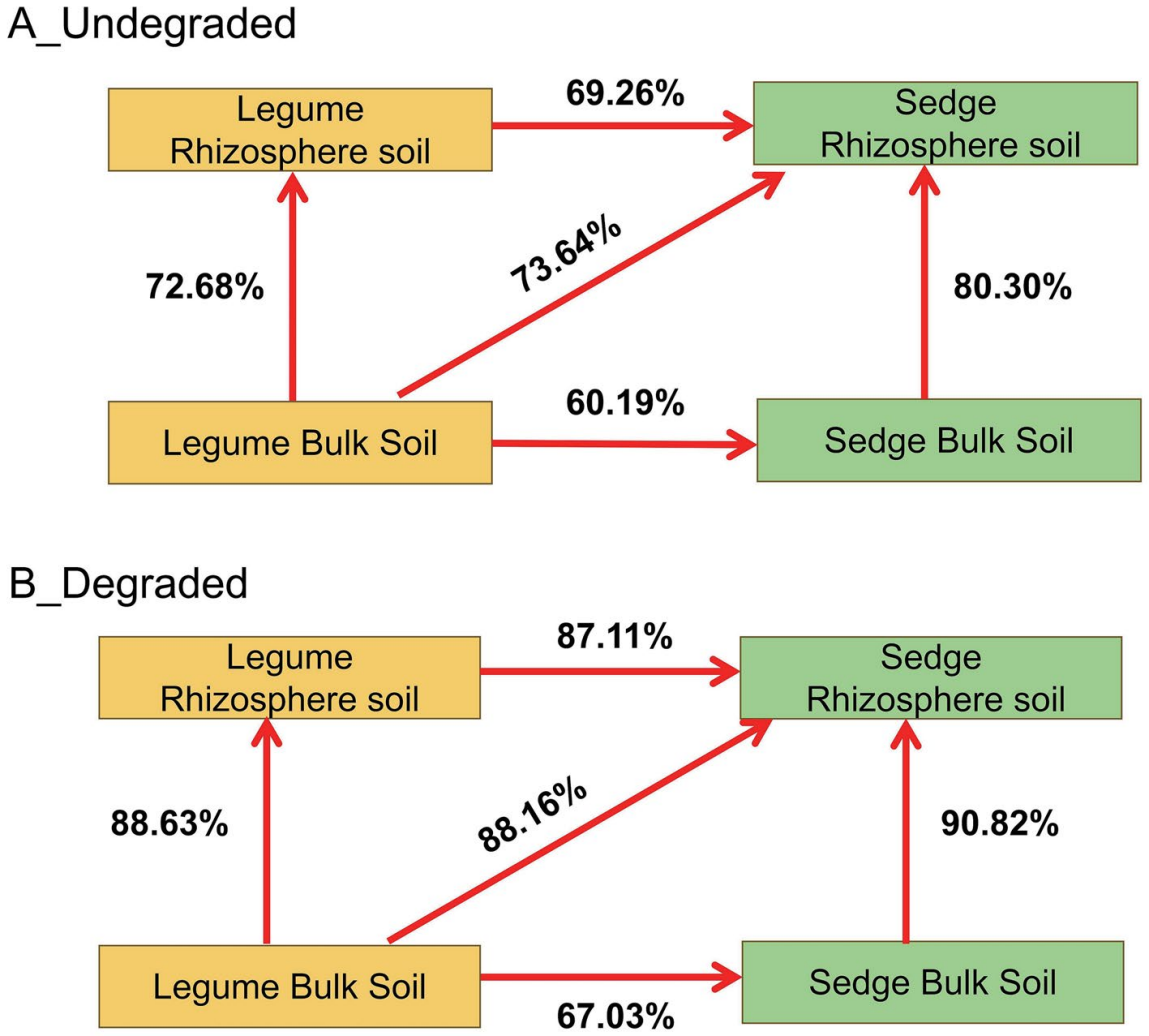
Sources of soil diazotrophs in sedge. **(A)** Sources of diazotrophs in the rhizosphere and bulk soils of sedge in the undegraded alpine meadow; **(B)** Sources of diazotrophs in the rhizosphere and bulk soils of sedge in the degraded alpine meadow.

To investigate the sources of diazotrophic communities in the rhizosphere and bulk soils of sedge during the degradation of alpine meadows, we used SourceTracker to analyze the similarity of these communities from legume (source) to sedge (sink). The results indicate, in undegraded meadows, 69.26% of the diazotrophs in the rhizosphere soil of sedge were derived from legume, while the corresponding proportion for the bulk soil was 60.19% (Figure 5A). In degraded meadows, 87.11 and 67.03% of the diazotrophs in the rhizosphere and bulk soils of sedge, respectively, were derived from legume (Figure 5B). In undegraded meadows, 72.68% of the diazotrophs in legume rhizosphere soil were derived from bulk soil, while the corresponding proportion for sedge rhizosphere soil was 80.30%. Under degraded conditions, 88.63% of the diazotrophs in legume rhizosphere soil were derived from bulk soil, while the corresponding proportion for sedge rhizosphere soil was 90.82%. In undegraded meadows, 73.64% of the diazotrophs in sedge rhizosphere soil were derived from the bulk soil of legume. In contrast, under degraded conditions, this proportion increased to 88.16%. These results suggest that meadow degradation increased the potential contribution of legume-soil diazotrophs to sedge soils, particularly in the rhizosphere. This pattern indicates that legume soils may act as an important diazotroph reservoir for sedge soils under degraded conditions. However, because SourceTracker infers source contributions based on community similarity, this recruitment pattern should be interpreted as putative rather than direct evidence of microbial transfer.

### 3.5 Diazotrophic community variation is associated with environmental factors and aboveground biomass

**Figure 6:**
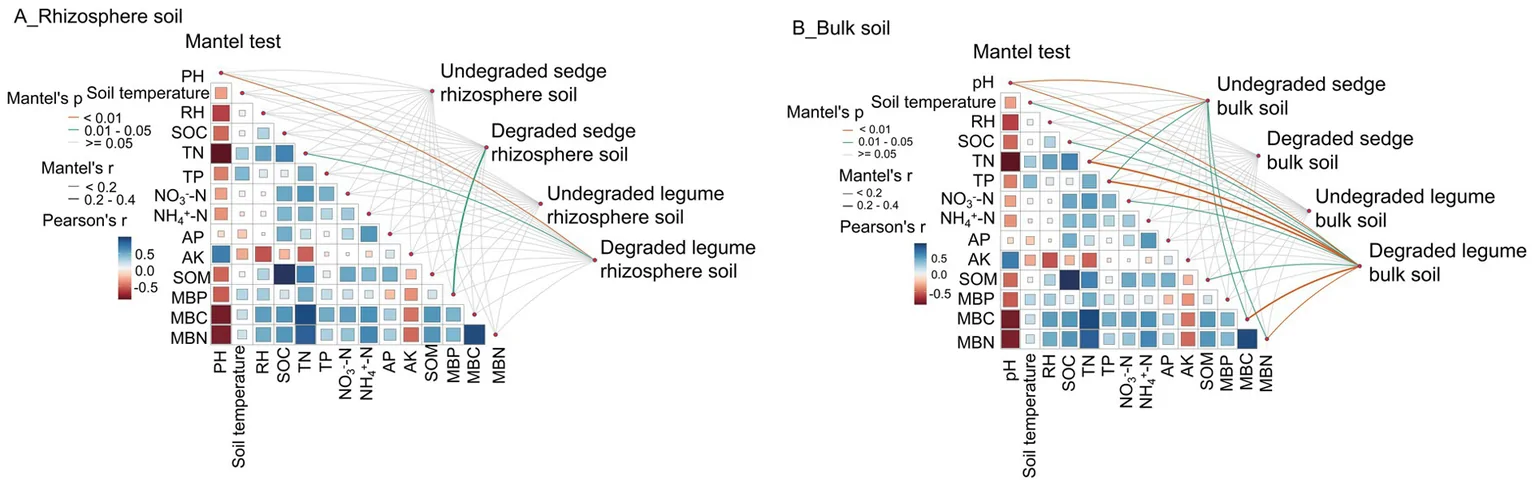
Mantel correlation analysis between diazotrophic communities and environmental factors. **(A)** Correlation between rhizosphere soil diazotrophic communities and environmental factors; **(B)** Correlation between bulk soil diazotrophic communities and environmental factors.

To identify the key environmental factors driving the variation in soil diazotrophic communities within degraded meadows, a Mantel test was performed to examine the relationships between the diazotrophs community and both aboveground plant factors and soil physicochemical properties. The results indicated that Soil temperature, SOC, TN, NH₄^+^-N, AP, and vegetation cover exerted highly significant effects on the variation of the diazotrophs community (Figures 6A,B). The exploratory PCA-based piecewise SEM showed an acceptable model fit. Meadow degradation significantly reduced soil nutrient PC1 and diazotroph *β*-diversity, whereas the paths from soil nutrient PC1 and vegetation diversity PC1 to aboveground biomass (AGB) were weak and non-significant (Supplementary Figure S2). These results indicate that AGB was not strongly mediated by the soil nutrient-diazotroph-vegetation diversity pathway.

## 4 Discussion

Soil microorganisms play essential roles in regulating biodiversity, nutrient cycling, and ecosystem functioning in grassland ecosystems (van der Heijden et al., 2008; Delgado-Baquerizo et al., 2016; Jansson and Hofmockel, 2020). Among them, diazotrophs represent a key functional group because of their ability to contribute biologically fixed nitrogen to nitrogen-limited soils. In this study, alpine meadow degradation significantly reduced the *α*-diversity of diazotrophic communities and altered their community composition, suggesting that degradation imposes strong environmental filtering on soil diazotrophs. Similar responses have been reported in degraded alpine wetlands, where degradation markedly changed the diversity and composition of diazotrophic communities (Li et al., 2022). These findings indicate that diazotrophic communities are sensitive to degradation-induced environmental changes in alpine ecosystems.

Proteobacteria was the dominant diazotrophic phylum in the present study, which is consistent with previous observations in grassland, forest, and agricultural soils (Jin et al., 2019). Notably, its relative abundance increased significantly in the rhizosphere and bulk soils of legume under degradation, whereas no significant change was observed in sedge soils. This plant-specific response may reflect differences in rhizosphere conditions, host-associated selection, and resource availability under degraded conditions (Naylor et al., 2017). Meadow degradation was accompanied by declines in soil moisture, SOC, TN, and vegetation cover, all of which may alter carbon and nutrient supply for diazotrophs. In addition, the enrichment of the asymbiotic diazotroph Skermanella in degraded meadows suggests that degradation may favor diazotrophic taxa with greater adaptability to nutrient-poor or disturbed soil environments. Together, these results suggest that alpine meadow degradation reshapes diazotrophic communities by modifying soil environmental conditions and plant-associated microbial habitats.

Alpine meadow degradation significantly altered soil nutrient status and vegetation cover, thereby reshaping diazotrophic community structure. Mantel test results showed that SOC, TN, vegetation cover, AP, soil temperature, and NH₄^+^-N were closely associated with diazotrophic community variation. In particular, the decline in SOC and TN may reduce carbon and nutrient availability for diazotrophs, strengthen environmental filtering, and contribute to the decrease in diazotrophic diversity under degradation. Aboveground biomass (AGB), used as a proxy for grassland productivity, was further evaluated using exploratory PCA-based piecewise SEM. Aboveground biomass is commonly regarded as an important indicator of community-level productivity in grassland ecosystems (Zhou et al., 2022; Zhao et al., 2022), and previous studies have shown close relationships among soil microbial diversity, vegetation diversity, soil properties, and ecosystem productivity (Delgado-Baquerizo et al., 2017). However, our SEM results indicated that degradation mainly affected soil nutrient status and diazotroph *β*-diversity, but provided limited evidence for direct diazotroph-mediated effects on AGB. Although diazotroph β-diversity was associated with vegetation diversity, the pathways from soil nutrient PC1 and vegetation diversity PC1 to AGB were weak and non-significant. Thus, the higher AGB observed in degraded plots may reflect short-term or stage-dependent vegetation responses, such as shifts in plant composition or compensatory growth. These results suggest that restoration should focus on rebuilding soil carbon and nitrogen pools, maintaining vegetation cover, and conserving legume-associated diazotroph reservoirs to support belowground microbial habitats and nitrogen cycling in degraded alpine meadows.

Understanding the ecological processes that govern microbial community assembly is essential for explaining microbial diversity and biogeographic patterns (Choudoir et al., 2016; Wu et al., 2019). Niche-based and neutral theories provide two complementary frameworks for interpreting community assembly. Niche-based theory emphasizes deterministic processes driven by environmental filtering and biotic interactions, whereas neutral theory highlights stochastic processes such as ecological drift, dispersal limitation, and random colonization (Chase and Myers, 2011). Increasing evidence suggests that microbial communities are not assembled by a single process, but by the joint effects of deterministic and stochastic mechanisms (Caruso et al., 2011; Dong et al., 2021; Riddley et al., 2025). Therefore, identifying the relative contributions of these processes is important for understanding how diazotrophic communities respond to alpine meadow degradation.

In this study, NST analysis indicated that diazotrophic communities associated with the legume species were mainly governed by deterministic processes, whereas those associated with the sedge species were more strongly influenced by stochastic processes. This contrast suggests that plant identity plays an important role in shaping diazotrophic community assembly. Legumes may impose stronger host selection on diazotrophs through root-associated niches and potential symbiotic associations, thereby enhancing deterministic filtering in both rhizosphere and bulk soils (Wang et al., 2025).

The iCAMP results further showed that the dominant assembly process in legume-associated soils shifted from dispersal limitation in undegraded meadows to homogeneous selection in degraded meadows. This shift indicates that degradation may strengthen environmental filtering, likely through changes in soil nutrient availability, moisture, and vegetation structure. For sedge-associated soils, homogeneous selection consistently dominated diazotrophic assembly in the rhizosphere, whereas stochastic processes such as dispersal limitation and ecological drift were more important in bulk soil. This pattern suggests that the sedge rhizosphere still provides a selective microhabitat for diazotrophs, even though sedge-associated diazotrophic communities were generally more stochastic than those associated with legumes. In contrast, bulk soils are less directly affected by host plants and may therefore be more susceptible to random dispersal and ecological drift. Together, these findings indicate that alpine meadow degradation does not simply shift diazotrophic assembly from deterministic to stochastic processes. Instead, its effects depend strongly on plant species and soil compartment, with legume-associated soils showing stronger deterministic filtering and sedge bulk soils showing greater stochasticity.

SourceTracker analysis suggested that sedge soils shared a large proportion of diazotrophic communities with legume soils, and this putative contribution increased under meadow degradation. In particular, the estimated contribution of legume soils to sedge rhizosphere diazotrophs increased from 69.26% in undegraded meadows to 87.11% in degraded meadows. This pattern indicates that legume soils may serve as important reservoirs of diazotrophs for sedge-associated soils, especially under degraded conditions. The plant-specific distribution of key diazotrophic taxa further supports this interpretation. Mesorhizobium was mainly enriched in the legume rhizosphere, where legume-rhizobia associations may provide a favorable niche, whereas its relative abundance was very low in sedge soils. Although sedges do not form root nodules, their rhizosphere diazotrophic communities showed high similarity to those associated with legumes. This may reflect shared soil microbial pools, rhizosphere habitat filtering, or potential microbial dispersal between neighboring plant-associated soils. However, further evidence from root exudate profiles, microbial activity assays, and spatially explicit sampling is needed to clarify the underlying mechanisms.

SourceTracker infers potential source contributions based on community similarity and should not be interpreted as direct evidence of microbial transfer. Future studies combining nifH gene abundance, nifH transcription, nitrogenase activity measurements, and ^15^N₂ incorporation would help verify the functional consequences of diazotroph recruitment and community reassembly in degraded alpine meadows.

## 5 Conclusion

This study demonstrates that alpine meadow degradation is associated with reduced diazotroph diversity and altered community composition in rhizosphere and bulk soils of legume and sedge. Diazotrophic communities in legume soils were mainly governed by deterministic processes, whereas those in sedge soils were more strongly influenced by stochastic processes, with rhizosphere-specific homogeneous selection observed in sedge. Environmental factors, including soil temperature, TN, SOC, NH4 + -N, AP, and vegetation cover, were closely related to diazotrophic community variation. Source-tracking analysis further suggested that legume soils may serve as an important diazotroph reservoir for sedge soils, and this potential contribution increased under degradation. Exploratory PCA-based SEM further showed that degradation significantly reduced soil nutrient status and diazotroph *β*-diversity, while the pathways from soil nutrient status and vegetation diversity to aboveground biomass were weak. These results suggest that the observed variation in aboveground biomass may be more closely associated with vegetation structural or compositional shifts than with direct mediation by diazotrophic communities or soil nutrients.

## Data Availability

The datasets presented in this study can be found in online repositories. The names of the repository/repositories and accession number(s) can be found at: https://ngdc.cncb.ac.cn/gsa/, CNCB.

## Footnotes

1
https://inap.denglab.org.cn
