# Supplementary material for: Alpine meadow degradation reshapes diazotroph community assembly and enhances putative recruitment of diazotrophs from legume soils to sedge soils
Source: Front Microbiol. 2026 Aug 19;17:1883674. doi: 10.3389/fmicb.2026.1883674 (PMC13536597; doi:10.3389/fmicb.2026.1883674)
Supplement: Supplementary file 1 [file Data_Sheet_1.docx]

**Supplementary Information**

# 1 Supplementary Data

## 1.1 Soil and plant sampling

Soil pH was measured using a pH meter (PHS-3C) with a soil-to-water ratio of 2.5:1. The total nitrogen (TN) content of the soil was determined using the Kjeldahl method. Soil organic matter (OM) content was determined by the potassium dichromate oxidation method with oil-bath heating. Soil nitrate‑nitrogen (NO_3_^-^-N) content was determined using the ultraviolet spectrophotometric method. Soil ammonium‑nitrogen (NH_4_^+^-N) content was measured by the indophenol blue colorimetric method. Soil available phosphorus (AP) content was determined by the sodium bicarbonate extraction–molybdenum blue colorimetric method (Olsen method). Soil available potassium (AK) content was measured using flame photometry(Bao 2006). Soil microbial biomass phosphorus (MBP), carbon (MBC), and nitrogen (MBN) were determined by the chloroform fumigation-extraction method(Wu *et al.* 2006).

Intact root systems with adhering soil, collected from the field, were subsequently processed in the laboratory. Bulk soil was defined as the soil shaken off from the roots, whereas rhizosphere soil was defined as the soil tightly adhering to the roots that could not be removed by shaking(Chaudhary *et al.* 2015). For each plant species, the intact plant sample was gently shaken to dislodge the loose soil from the roots. The soil that fell off was collected as the bulk soil. This process was repeated until no more soil could be dislodged. The roots were washed by adding 30 mL of sterile PBS buffer (pH 7.0, with 0.1% Tween-80) into a 50 mL sterile centrifuge tube containing the roots, followed by hand inversion for thorough immersion and ultrasonic treatment for 10 minutes. The wash solution was then transferred to a fresh sterile tube. This process was performed in triplicate. The solution was centrifuged at 6000 × g for 5 min. The resulting pellet (soil) was then freeze-dried in a lyophilizer at -40℃ for at least 12 hours. The resulting dried soil was defined as the rhizosphere soil. Both types of soil (rhizosphere and bulk) were then stored at -80℃ until DNA extraction.

## 2 Supplementary Figures and Tables

**Supplementary Table 1. Genus-level abundance of diazotrophs across different treatments.**

| Sample | Genus | Significance | Sample | Genus | Significance |
| --- | --- | --- | --- | --- | --- |
| BD1 VS BS1 | *Skermanella* | b | RS1 VS RD1 | *Skermanella* | a |
|  | *Skermanella* | a |  | *Skermanella* | b |
|  | *Nostoc* | a |  | *Nostoc* | a |
|  | *Nostoc* | a |  | *Nostoc* | b |
|  | *Mesorhizobium* | a |  | *Mesorhizobium* | b |
|  | *Mesorhizobium* | b |  | *Mesorhizobium* | a |
|  | *Frankia* | a |  | *Frankia* | b |
|  | *Frankia* | b |  | *Frankia* | a |
|  | *Paenibacillus* | a |  | *Paenibacillus* | a |
|  | *Paenibacillus* | a |  | *Paenibacillus* | a |
|  | *Geobacter* | a |  | *Geobacter* | a |
|  | *Geobacter* | a |  | *Geobacter* | a |
| BD2 VS BS2 | *Skermanella* | a | RS2 VS BS2 | *Skermanella* | a |
|  | *Skermanella* | a |  | *Skermanella* | b |
|  | *Nostoc* | a |  | *Nostoc* | a |
|  | *Nostoc* | a |  | *Nostoc* | a |
|  | *Mesorhizobium* | a |  | *Mesorhizobium* | a |
|  | *Mesorhizobium* | a |  | *Mesorhizobium* | a |
|  | *Frankia* | a |  | *Frankia* | a |
|  | *Frankia* | a |  | *Frankia* | a |
|  | *Paenibacillus* | a |  | *Paenibacillus* | a |
|  | *Paenibacillus* | a |  | *Paenibacillus* | a |
|  | *Geobacter* | a |  | *Geobacter* | a |
|  | *Geobacter* | a |  | *Geobacter* | a |
| BD1 VS RD1 | *Skermanella* | a | RS2 VS RD2 | *Skermanella* | a |
|  | *Skermanella* | a |  | *Skermanella* | a |
|  | *Nostoc* | a |  | *Nostoc* | a |
|  | *Nostoc* | a |  | *Nostoc* | a |
|  | *Mesorhizobium* | b |  | *Mesorhizobium* | a |
|  | *Mesorhizobium* | a |  | *Mesorhizobium* | a |
|  | *Frankia* | a |  | *Frankia* | a |
|  | *Frankia* | a |  | *Frankia* | a |
|  | *Paenibacillus* | a |  | *Paenibacillus* | a |
|  | *Paenibacillus* | a |  | *Paenibacillus* | a |
|  | *Geobacter* | a |  | *Geobacter* | a |
|  | *Geobacter* | a |  | *Geobacter* | a |
| BD2 VS RD2 | *Skermanella* | a | BD1 VS BD2 | *Skermanella* | b |
|  | *Skermanella* | a |  | *Skermanella* | a |
|  | *Nostoc* | a |  | *Nostoc* | a |
|  | *Nostoc* | a |  | *Nostoc* | a |
|  | *Mesorhizobium* | a |  | *Mesorhizobium* | a |
|  | *Mesorhizobium* | a |  | *Mesorhizobium* | a |
|  | *Frankia* | a |  | *Frankia* | a |
|  | *Frankia* | a |  | *Frankia* | a |
|  | *Paenibacillus* | a |  | *Paenibacillus* | a |
|  | *Paenibacillus* | a |  | *Paenibacillus* | a |
|  | *Geobacter* | a |  | *Geobacter* | a |
|  | *Geobacter* | b |  | *Geobacter* | a |
| RS1 VS BS1 | *Skermanella* | a | BS1 VS BS2 | *Skermanella* | a |
|  | *Skermanella* | a |  | *Skermanella* | a |
|  | *Nostoc* | a |  | *Nostoc* | a |
|  | *Nostoc* | a |  | *Nostoc* | a |
|  | *Mesorhizobium* | a |  | *Mesorhizobium* | a |
|  | *Mesorhizobium* | a |  | *Mesorhizobium* | a |
|  | *Frankia* | a |  | *Frankia* | a |
|  | *Frankia* | a |  | *Frankia* | a |
|  | *Paenibacillus* | a |  | *Paenibacillus* | a |
|  | *Paenibacillus* | a |  | *Paenibacillus* | a |
|  | *Geobacter* | a |  | *Geobacter* | a |
|  | *Geobacter* | a |  | *Geobacter* | a |
| RD1 VS RD2 | *Skermanella* | b | RS1 VS RS2 | *Skermanella* | b |
|  | *Skermanella* | a |  | *Skermanella* | a |
|  | *Nostoc* | b |  | *Nostoc* | a |
|  | *Nostoc* | a |  | *Nostoc* | a |
|  | *Mesorhizobium* | a |  | *Mesorhizobium* | a |
|  | *Mesorhizobium* | a |  | *Mesorhizobium* | a |
|  | *Frankia* | a |  | *Frankia* | a |
|  | *Frankia* | b |  | *Frankia* | a |
|  | *Paenibacillus* | a |  | *Paenibacillus* | a |
|  | *Paenibacillus* | a |  | *Paenibacillus* | a |
|  | *Geobacter* | a |  | *Geobacter* | a |
|  | *Geobacter* | a |  | *Geobacter* | a |

Note: 1:Undegraded 2:Degraded B: Bulk soil; R: Rhizosphere soil; D: legume; S: sedge.

**Supplementary Table 2. PERMANOVA results showing the effects of different factors on diazotrophic community variation.**

| Factor | R^2^ | F | P |
| --- | --- | --- | --- |
| Degradation status | 0.09 | 10.44 | 0.0001 |
| Plant type | 0.027 | 3.165 | 0.002 |
| Soil compartment | 0.016 | 1.832 | 0.0391 |

**Supplementary Figure 1. Abundance of the main genus of diazotrophs of legume and sedge.**

**Supplementary Figure 2.**

**Exploratory PCA-based piecewise structural equation models showing the direct and indirect relationships among alpine meadow degradation, soil nutrient status, diazotroph β-diversity, vegetation diversity, and aboveground biomass (AGB) in legume rhizosphere soil (A) and sedge rhizosphere soil (B)**.

Note: Solid arrows indicate significant paths, dashed arrows indicate non-significant paths, black arrows indicate positive effects, and blue arrows indicate negative effects. Numbers beside arrows represent standardized path coefficients.
